# Sensitivity-Improved Ultrasonic Sensor for 3D Imaging of Seismic Physical Model Using a Compact Microcavity

**DOI:** 10.3390/s18072315

**Published:** 2018-07-17

**Authors:** Tingting Gang, Manli Hu, Xiaohong Bai, Qiangzhou Rong

**Affiliations:** Physics Department, Northwest University, No. 229, Taibai Road (North), Xi’an 710069, China; tingtinggang1@163.com (T.G.); baixiaohonglxl@163.com (X.B.)

**Keywords:** Fabry–Perot interferometer, ultrasonic detection, ultrasonic imaging

## Abstract

A sensitivity-improved ultrasonic sensor is proposed and demonstrated experimentally in this present study. The device is comprised only a fiber-optic microcavity that is formed by discharging a short section of hollow core fiber (HCF). The key to ensuring the success of the sensor relies on the preprocessing of hydrogen loading for HCF. When discharging the HCF, the hydrogen is heated up during the formation of the air bubble, which enlarges the bubble diameter, smoothens its surfaces simultaneously and decreases Young’s modulus of the material of the bubble. Ultimately, this results in the probe being highly sensitive to ultrasound with a SNR of 69.28 dB. Once the compact air cavity is formed between the end face of the leading-in fiber and the top wall of the bubble, a well-defined interference spectrum is achieved based on the Fabry–Perot interference. By using spectral side-band filtering technology, we detect the ultrasonic waves reflected by the seismic physical model (SMF) and then reconstruct its three-dimensional image.

## 1. Introduction

As a hot topic in the international research community, the fiber-optic ultrasonic detecting technique can be used as an effective method for evaluating the microstructure and related mechanical properties and for detecting the microscopic and macroscopic discontinuities of solid materials [1]. This technique that perceives the ultrasonic-related information is implemented by detecting the light intensity, wavelength, phase, polarization and other parameters of transmission light in fibers. Fiber-optic ultrasonic sensors have many advantages [2,3,4] compared with traditional electrical ultrasonic transducers, including a capacity to detect broad-band acoustic wave signals with high sensitivity and resist disturbance; good reusability; and improvement of the reliability and efficiency of ultrasonic detection. All these advantages are beneficial to improve the reliability and efficiency of imaging of seismic physical models (SPMs) [5]. The SPM is used to simulate the real geological structure in laboratories, which can set up a bridge between theories and field-scale experiments. Compared with the experiments in the field, this is more cost-effective and more repeatable compared to the experiments in the field. Currently, the ultrasonic sensors are divided into two main categories [6]: fiber-optic interferometers and fiber gratings. These two types of sensors have unique superiority in different practical application fields. Ultrasonic sensors based on fiber grating generally employ three different types of gratings as the sensing units, which includes fiber Bragg grating (FBG) [7], phase-shifted fiber Bragg grating (PI–FBG) [8] and FBG based on Fabry–Perot interferometer (FPI–FBG) [9]. These devices can be utilized to implement a sensor network of multiple gratings in a single fiber because of their narrow bandwidth and ease in multiplexing. In general, the spectral side-band filter technique is used in the signal demodulation of fiber-optic ultrasonic sensors [10,11], where an increase in the spectral slope leads to a higher sensitivity. The length of the grating should be as large as possible to increase the spectral slope for the FBG ultrasonic probe. However, when the length of the grating is larger than the ultrasonic wavelength, the sensitivity of the sensor to detect ultrasonic waves (UWs) will reduce dramatically [12], which hardly meets the requirements of ultrasonic imaging of seismic physics SPMs. According to the interference mechanism, fiber-optic interferometric ultrasonic sensors are commonly based on Mach–Zehnder interference (MZI) [13], Fabry–Perot interference (FPI) [14], Michelson interference (MI) [15] and Sagnac interference (SI) [16]. Among these, FPI is the most widely used interference for ultrasonic detection and imaging, which has the main advantages of detecting distance, signal-noise ratio (SNR) and response frequency band (a more compact structure) [17,18,19]. An FPI created between a fiber end face and a reflective diaphragm is one of the most common sensor configurations [20]. Generally, the reflective diaphragm uses an elastic diaphragm at the fiber tip, such as SiO_2_/silica, polymer, silver and graphene film. An ultrasonic pressure applied to the FP-based interferometer [21] will introduce a strain along the fiber, stretching the cavity periodically.

Considering the practical applications of ultrasonic sensors in imaging (large attenuation, absorption and scattering of UWs during complicated geological structure transmission), it is necessary to focus on the improvement of the ultrasonic sensitivity. The pressure sensitivity of the diaphragm-based fiber tip FP-based sensor is defined as the ratio of the FP cavity length variation to the pressure change, which critically depends on the size and the mechanical quality of the diaphragm employed [22]. Bing Sun proposed an ultracompact polymer-capped FP interferometer with a cavity length of 35.1 µm and demonstrated that the pressure sensitivity was 1130 pm/MPa [23]. A fiber-tip pressure sensor has been reported with a pressure sensitivity of 1.49 nm/psi (1 psi = 6.89 kPa), which was made by splicing a 66-μm-diameter and 1.88-μm-thick silica diaphragm to a microcavity at an optical fiber end [24]. Feng Xu presented an EFPI pressure sensor using a 130-nm-thick and 125-μm-diameter silver diaphragm with a pressure sensitivity of 70.5 nm/KPa [25]. From previous reports, it is obvious that the most effective method to improve the pressure sensitivity is to reduce the thickness of the diaphragm as much as possible. However, the improvement of the sensitivity is restricted because of fragile mechanical properties of diaphragms that are much too thin.

In this paper, we propose and experimentally demonstrate a novel sensitivity-improved ultrasonic sensor based on an all-silica FPI (similar to a microcavity). The sensor is made by discharging a short section of HCF with preprocessing of hydrogen loading. During the process of discharging, the hydrogen is heated up during the formation of air bubbles, which enlarges the bubble diameter (185 μm) and smoothens its surfaces simultaneously. Furthermore, the interaction between hydrogen bond and silicon dioxide leads to a wall with a small Young’s modulus. Therefore, the proposed sensor exhibits a high pressure sensitivity of ~3600 pm/MPa, which is two times higher than the our previous result (without the operation of hydrogen loading) [26]. From the results of ultrasonic detection and 3D imaging, the proposed sensor is recognized to have potential applications in the determination of complex geological structures under the premise of further data optimization.

## 2. Sensor Fabrication and Operation Principle

### 2.1. Sensor Fabrication

**Figure 1:**
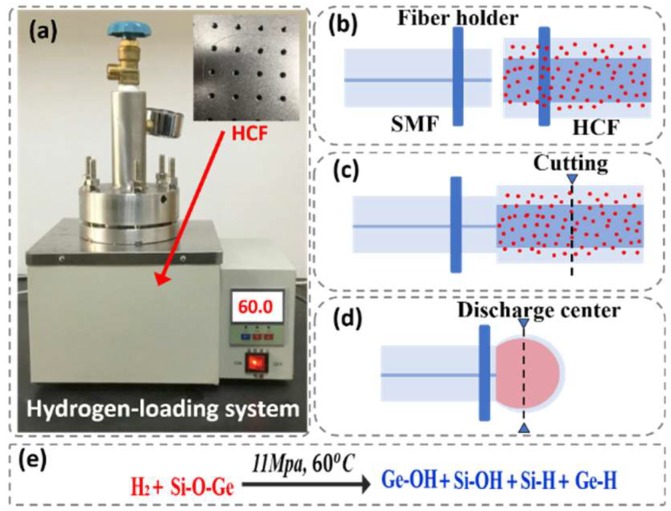
(**a**–**d**) Fabrication process of the proposed sensor and (**e**) equation.

**Figure 2:**
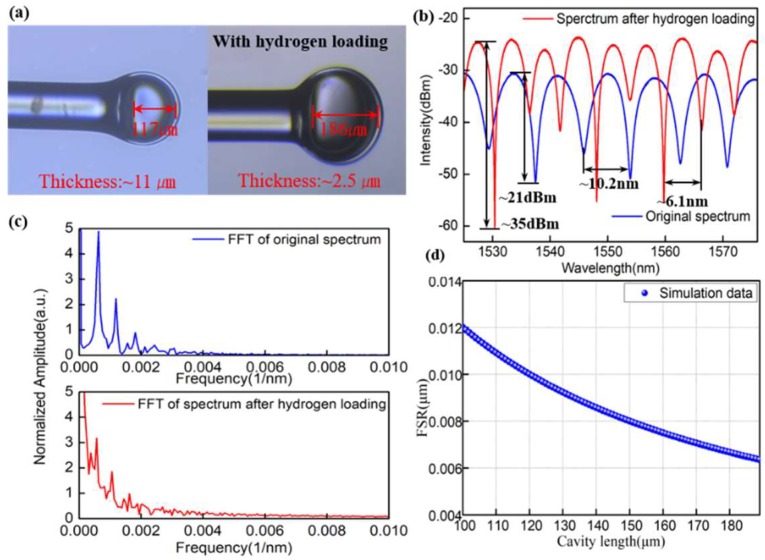
Without hydrogen loading and after hydrogen loading: (**a**) photographs of microfiber cavity; (**b**) spectrograms; (**c**) spatial frequency and (**d**) theoretical calculation on FSR as function of cavity length.

Figure 1 shows the fabrication process of the proposed FPI-based sensor, which consists of four steps. In Step 1, a section of HCF was stored in the hydrogen chamber shown in Figure 1a at a pressure of 11 MPa and temperature of 60 °C for 14 days. During the process, the hydrogen molecular (H_2_) could be diffused into the HCF gradually, which formed Ge-OH, Si-OH, Si-H and Ge-H (shown in Figure 1e). The next step involves a section of SMF and HCF (the diameters of hollow core and cladding were 100 µm and 250 µm, respectively) with cleaved ends being placed in the left and right holds of a Fujikura arc fusion splicer, respectively (Figure 1b). After this, the HCF was cleaved under a microscope and the final length of HCF was 250 µm. In the final step, the end face of HCF was placed in the left or right hold of the Fujikura arc fusion splicer and the end face of HCF was constantly discharged with the power of −40 bits for the time of 4500 ms until the formation of a thin micro bubble (shown in Figure 2d). During the process of discharging, the hydrogen molecules in the HCF were heated up and expanded, thus resulting in a bubble with a large size, thin thickness and smooth surfaces compared with those reported in previous reports [26] (This is also a highlight of this work). The length and thickness of the microbubble were about 186 µm and 2 µm, respectively, as shown in Figure 2a. Once the device is formed, a well-defined interference spectrum is achieved using the FPI. Figure 2b shows the reflection spectra of the sensor without hydrogen loading and with hydrogen loading in the wavelength range of 1510–1610 nm under standard atmospheric pressure at room temperature. In comparison, the spectrum of the proposed sensor was optimized dramatically in terms of the extinction ratio (EI; from 21 dBm to 35 dBm) and the interference fringe, while the main interference pattern could be modified further because of the improved smoothness of the bubble. In addition, the increase in cavity length resulted in a decrease in the free spectral range (FSR) from 10.2 nm to 6.1 nm. This result agrees with the theoretical calculation on FSR as a function of cavity length shown in Figure 2d. To further analyze the characteristics of the two interference patterns, the wavelength spectra in Figure 2b were Fourier transformed to the spatial frequency, as shown in Figure 2c. It is clearly seen that the generation of these interference spectra is due to the reflection of core modes by the end-face of SMF and of the low-order cladding modes. Besides, a part of the light may be reflected back to the leading-in SMF by the outside surface of the bubble cavity. All of these results were shown in the inhomogeneous interference patterns.

### 2.2. Operation Principle

Assuming that illumination has a flat broadband spectrum and the refractive index of gas in the bubble is very close to 1, the output spectral intensity (*I*) of sensor is [27]:

(1) I=K×[A12+A22+2A1A2cos(4πdλ)],

where K is a constant; $A_{1}$ and $A_{2}$ are the electric field amplitudes of the two interfering interfaces; λ represents wavelength of the light source and d is the cavity length.

**Figure 3:**
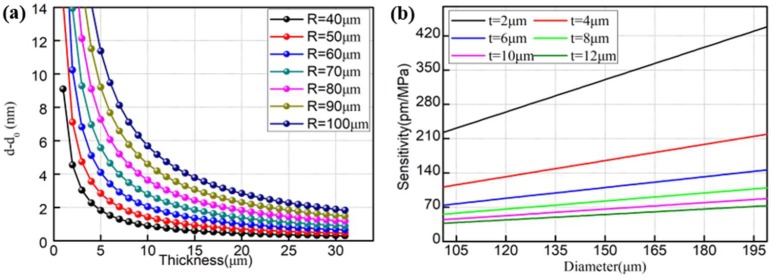
Theoretical calculations on (**a**) cavity length change versus the bubble thickness and (**b**) pressure sensitivity as a function of bubble thickness under different cavity lengths.

When an ultrasonic pressure is applied on the proposed sensor, the wavelength shift would occur as follows, which was described by reference [27]:

(2) $$∆\lambda =\frac{\lambda ∆d}{d},$$

where ∆d is the variation of the air-cavity length caused by the pressure-induced deflection of silica wall. If we assume that the pressure on the bubble is isotropic, this variable can be expressed as follows [27]:

(3) $$∆d=\frac{\left(1-v\right)R^{2}∆P}{2Et},$$

where v, E, R, t and ∆p are the Poisson’s ratio, Young’s modulus of silica, the radius and thickness of the bubble and change in pressure, respectively. For fused silica, E = 73 GPa and v = 0.17. Figure 3a shows the calculated relationship between the thickness of the bubble and the variation in cavity length under gas pressure at the cavity radius of 40–100 µm. It can be clearly seen that the variation in cavity length increases with the increase in cavity radius, while it decreases when the silica wall thickness decreases. It is worth noting that the variation in cavity length increases significantly when the thickness of the bubble wall is less than 5 µm.

The sensitivity to pressure can be defined by the interference wavelength shift (∆λ) caused by the variation in cavity length, which was given by reference [28] as:

(4) $$\frac{∆\lambda }{∆P}=\frac{\lambda d\left(1-v\right)}{4Et},$$

where *d* and λ are the diameter of the bubble and 1550 nm, respectively. Figure 3b shows the variation sensitivity with the increase in diameter of the fabricated bubble at thicknesses of 2 μm, 4 μm, 6 μm, 8 μm, 10 μm and 12 μm. According to the simulation results, the sensitivity to pressure is better for the bubble with larger diameter when the same wall thickness is used. Essentially, the method of obtaining high pressure sensitivity involves reducing the thickness and increasing the diameter of the sensor. The sensitivity of proposed sensor is ~360 pm/MPa, which is not high for pressure sensing. However, in practical applications, the device may be implemented by increasing the diameter because of the requirement of a stable and accurate control of the discharge current and time and weak mechanical strength of the sensor with thin thickness. Nonetheless, all these parameters depend on specific sensing requirements.

## 3. Pressure Measurement and Discussion

### 3.1. Experimental Setup for Measuring Pressure

**Figure 4:**
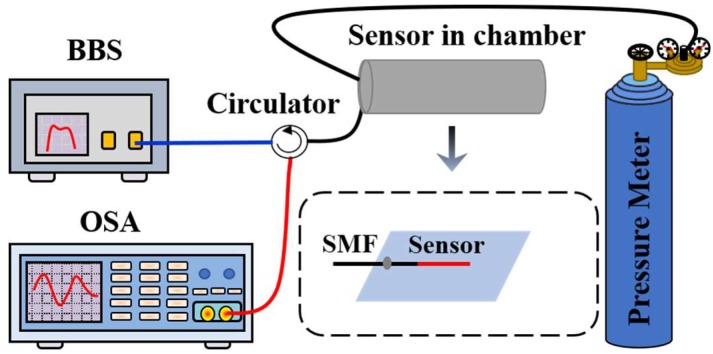
Experimental setup for measuring the response of the proposed sensor to the gas pressure.

Figure 4 shows the experimental setup of the proposed fiber-tip pressure sensor. The light from a broadband source (BBS) centered at 1550 nm was propagated to the sensing probe through an optical circulator. After this, it was reflected and guided to an optical spectrum analyzer (OSA) with a resolution of 0.02 nm by the optical circulator. The sensor head was placed into the gas chamber, where a commercial gas pressure generator was incorporated with a high-precision pressure meter to measure the pressure in the chamber. Furthermore, its pigtail fiber and the feed throat of the gas chamber were sealed by strong glue. When the pressure was increased to 1 MPa at increments of 0.1 MPa, the reflection spectrum of the sensor head was monitored using the OSA. The pressure was kept constant for 20 min to ensure a well-distributed pressure around the interferometer before each measurement.

### 3.2. Experimental Results

**Figure 5:**
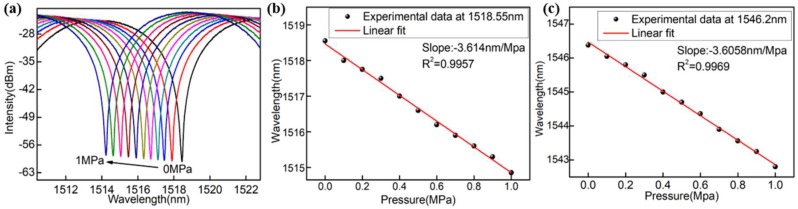
(**a**) Response of the micro-bubble sensor to pressure; resonant wavelengths of the interference fringe versus different gas pressures for the sensor at (**b**) 1518.55 nm and (**c**) 1546.2 nm.

As the gas pressure is gradually increased from 0 to 1 MPa, the interference spectrum presents a blue-shift with a slight fringe contrast fluctuation in Figure 5a. As shown in Figure 5b,c, the wavelength shifts of two different resonant dips (1518.55 nm and 1546.2 nm) versus different pressures were recorded. It was clearly found that the resonant wavelengths shifted linearly toward a shorter wavelength (blue-shift) as the applied gas pressure increased from 0 MPa to 1 MPa. The pressure sensitivities of the two resonant dips (slope of the fitting line) were 3614 pm/MPa and 3605.8 pm/MPa, which are both two orders of magnitude higher than that reported in our previous work. However, there is an obvious gap between the simulation results and experimental results. This is mainly because a certain reaction occurs between hydroxyl in HCF and silicon dioxide during the process of charging continually, resulting the material of the bubble (walls) having a smaller Young’s modulus.

## 4. Ultrasonic Detection, Imaging and Discussion

### 4.1. Experimental System for Ultrasonic Detection and Imaging

**Figure 6:**
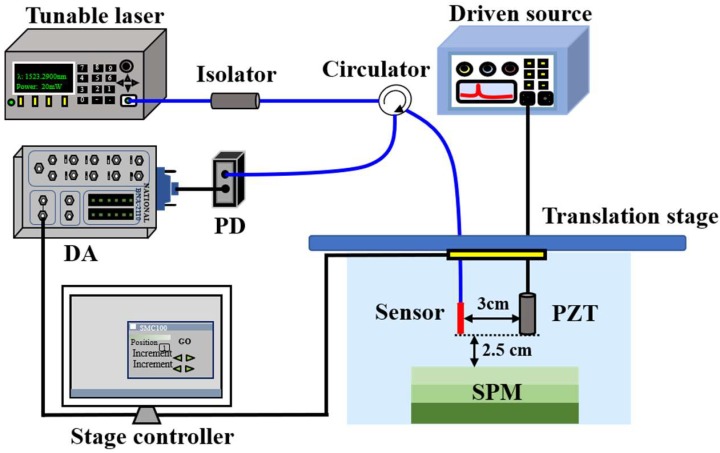
Experimental setup for UW detection and imaging.

Figure 6 shows the experimental setup for UW detection and imaging. A Piezoelectric transducer (PZT) with the resonant frequency of 1 MHz (Olympus 2077PR, Tokyo, Japan, diameter: 20 mm) driven by a pulse square wave or continuous sinusoidal wave was used to generate ultrasounds. The optical fiber sensor was illuminated by a tunable laser (Santec, TSL-710, Nagoya, Japan) with a line width of 100 kHz and power of 20 mW. The light was propagated to the sensing probe through the optical circulator. The wavelength shift caused by ultrasonic waves was transformed to the output power and eventually, the detection signal was recorded by a data acquisition card for further analysis. In the experiments, the edge filter technique was employed in the system. To improve the sensitivity, the laser was locked to the 3-dB position of one linear side of the interference fringe (1518.258 nm in our experiments). For comparison, in the experiments, we fabricated two sensors using ordinary HCF and HCF after hydrogen loading. The sensors placed in the water tank were exactly opposite to the PZT, while the PZT and sensor were fixed at a same translation stage at a distance of 3 cm.

### 4.2. Ultrasonic Detection

**Figure 7:**
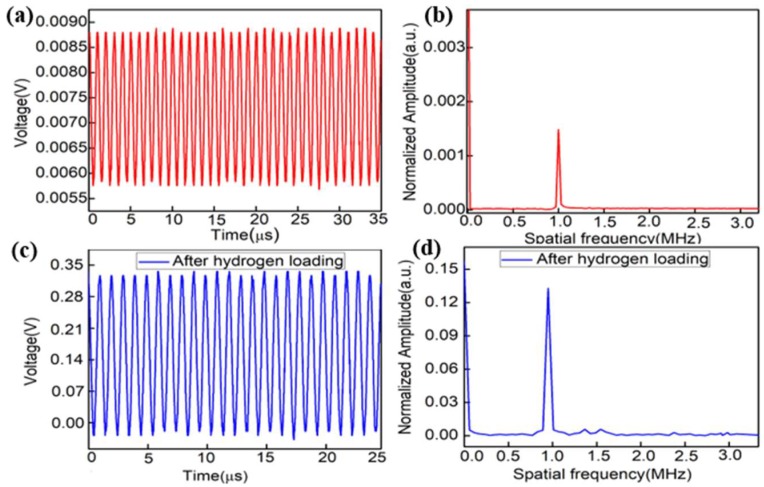
Response to (**a**) a 1-MHz continuous sinusoidal signal without hydrogen loading and (**b**) frequency spectrum; (**c**) a 1-MHz continuous sinusoidal signal with hydrogen loading and (**d**) frequency spectrum.

As shown in Figure 7a,c, a continuous sinusoidal ultrasound with the frequency of 1 MHz was detected effectively by the two sensors. Figure 7b,d shows the frequency domain spectra of a continuous UW calculated by the Fourier transformation, which depends on the frequency band and the resonance frequency of the PZT. The result verifies that both the fiber sensors have a good response to the ultrasonic signal with the frequency of 1 MHz.

**Figure 8:**
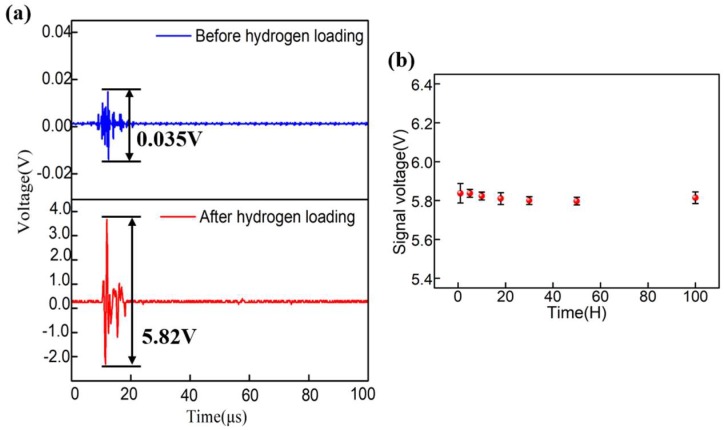
(**a**) Response to a 1-MHz pulse acoustic wave of sensor and (**b**) stability test of the sensor.

To study the layered property of the sensor further, the sensors’ response to a 1-MHz UW pulse (the ultrasonic frequency was also applied to the subsequent experiments of SPM imaging) is shown in Figure 8a. It is clearly seen that the peak-to-peak voltages of the detection signal using the two sensors were 0.035 V and 5.82 V, respectively. The sensitivity of the sensor fabricated by using the HCF with hydrogen loading was significantly increased, which is at least two orders of magnitude higher than that of the previous sensor based on HCF without hydrogen loading (it is 0.035 V as shown in the upper part of Figure 8a). Figure 8a shows the presence of other noises near the center response due to the inherent bandwidth of the PZT and low-frequency noises. All these unwanted noises were filtered out when the scanning images were reconstructed as described in the following section. Before imaging the SPM, it is necessary to detect the stability of the sensor, which is an important factor for practical applications. The measurement of the stability of the sensor was repeated five times to ensure its accuracy. Figure 8b shows the response peak-to-peak voltage fluctuations of the proposed sensor over 100 h, in which the red points represent the average response peak-to-peak voltage of these measurements and the black whiskers are the standard deviations. It is quite remarkable that the value remained almost constant (~5.82 V) during these hours. Although the maximum fluctuation of the ultrasonic response was 0.04 V, there was only a response voltage variation of 6%. This demonstrates that the proposed device is stable during the whole imaging process.

### 4.3. Seismic Physical Model Imaging

**Figure 9:**
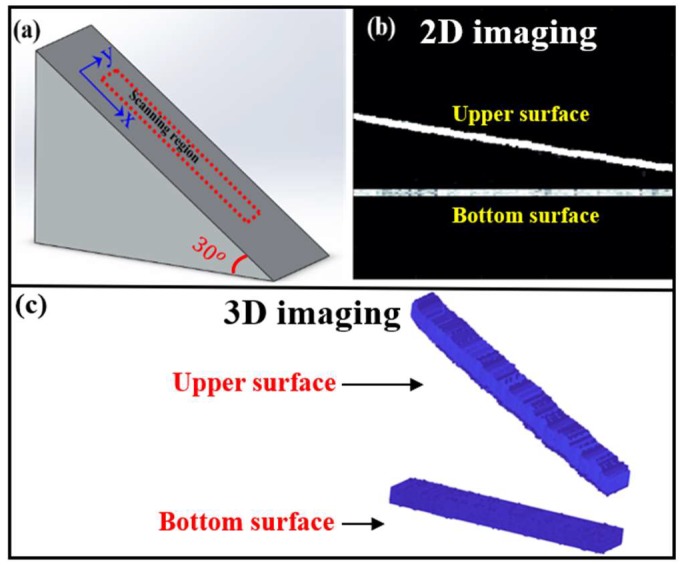
(**a**) Physical model, (**b**) lateral 2D image of the physical model and (**c**) 3D image of a certain region of the physical model.

The capacity of the sensors in SPM imaging is demonstrated as follows. The tested model, which simulates a geological dipping structure, is a slope with an angle of 30° as shown in Figure 9a and the region of the red dotted line is an imaging area with the size of 2.5 cm × 15 cm. In the experiments, the PZT source and fiber sensor were moved quasi-continuously, which were driven by the electric-driven stage with a step of 0.1 mm and the scanning direction was along the x- and y-axes in sequence. During the whole scanning process, the surrounding temperature was almost constant in the detected media of water due to the large specific heat capacity of water (4200 J/kg). Figure 9b shows the lateral 2D image (along the x-axis) of the SPM using the proposed sensor. We can clearly see a high contrast between the two surfaces of the SPM due to the good ultrasonic response of the proposed sensor. If we know the ultrasonic propagation velocity (1450 m/s) and frequency (1 MHz), the axial resolution of the sensor can be calculated to be 0.725 mm in water. Using ultrasounds with ultra-high frequency can further enhance the resolution of the system when the detected depth decreases, which is best applied to intra-corporal detection and imaging in a small space. Therefore, the choice of ultrasonic frequency (axial resolution) depends on the needs of a specific application. By scanning along the x- and y-axes repeatedly and data reconstruction, the 3D image of the scanning region can be obtained, as shown in Figure 9c, which is consistent with the shape of the model and the actual situation. The inclined plane and the bottom plane can be obviously seen. In conclusion, the proposed sensor has the ability to conduct 3D imaging of SPM. With further data optimization, the proposed sensor can be applied to complex SPMs.

### 4.4. Discussion

#### 4.4.1. Signal Noise Ratio (SNR)

The SNR is used to characterize the UW responses of the sensor. The SNR is highly related to the initial noise of experimental setups (especially caused by the power fluctuations of laser source, transmission line and sensor stability). The SNRs of the sensors are calculated by the following equation:

(5) $$\mathrm{SNR}=20log_{10}\frac{V_{pp}}{V_{n}},$$

where *Vn* is the noise level appearing before the ultrasonic response and *Vpp* is the peak-to-peak voltage of the initial response (2 mV for our set up). Thus, if we know the signal peak-to-peak voltage of 5.82 V (found in Figure 8a of the manuscript), the SNR of the proposed sensor is calculated as 69.28 dB. The SNR of the proposed sensor is much larger than SNR of the microbubble sensor without hydrogen loading (24.86 dB) [26]. The excellent feature makes the proposed sensor a good candidate for the imaging of SPMs.

#### 4.4.2. Resolution

The axial resolution of imaging depends on the ultrasonic velocity in a medium and the pulse duration, which can be expressed as [29]:

(6) $$R=\frac{\gamma \tau }{2},$$

where γ is the speed in a medium and τ is the ultrasonic pulse duration. Knowing the ultrasonic propagation velocity (1450 m/s) in water and ultrasonic frequency (1 MHz) allowed us to calculate the pulse duration of 1 μs in the experiment and the axial resolution of 0.725 mm in water. The resolution would change to 1.35 mm when the ultrasonic wave is transmitted in the SPMs (transmission speed: 2700 m/s).

## 5. Conclusions

In conclusion, we have demonstrated a simple fiber-optic micro FP interferometer for ultrasonic detection and imaging with ultra-high sensitivity (SNR: 69.28 dB). By splicing the HCF-loaded hydrogen with an SMF and discharging the HCF, a microbubble with a large diameter and thin thickness is naturally formed and exhibits a pressure sensitivity of ~3600 pm/MPa. In the experiments of ultrasonic detection and imaging, the spectral band-side filter technique is used for interrogation. The 3D images of SPMs were reconstructed by the proposed ultrasonic scanning system. The proposed sensor probe has an excellent performance using the compact structure with hydrogen loading, which can be a candidate for imaging of the real underlying structure.

## References

[1] Guo H., Xiao G., Mzih N., Yao J. (2011). Fiber Optic Sensors for Structural Health Monitoring of Air Platforms. Sensors.

[2] Rong Q., Hao Y., Zhou R., Yi X., Shao Z., Liang L., Qiao X. (2017). UW Imaging of Seismic-Physical-Models in Air Using Fiber-Optic Fabry–Perot Interferometer. Sensors.

[3] Zhang W., Wang R., Rong Q., Qiao X., Guo T., Shao Z., Li J., Ma W. (2017). An Optical Fiber Fabry–Perot Interferometric Sensor Based on Functionalized Diaphragm for Ultrasound Detection and Imaging. IEEE Photonics J..

[4] Rong Q., Shao Z., Yin X., Gang T., Liu F., Sun A., Qiao X. (2016). Ultrasonic Imaging of Seismic Physical Models Using Fiber Bragg Grating Fabry–Perot Probe. IEEE J. Sel. Top. Quantum Electron..

[5] Rong Q., Zhou R., Hao Y., Yin X., Shao Z., Gang T., Qiao X. (2017). Ultrasonic sensitivity-improved Fabry–Perot interferometer using acoustic focusing and its application for non-contact imaging. IEEE Photonics J..

[6] Gang T., Hu M., Qiao X., Li J., Shao Z., Tong R., Rong Q. (2017). Fiber-optic Michelson interferometer fixed in a tilted tube for direction-dependent ultrasonic detection. Opt. Lasers Eng..

[7] Tsuda H., Sato E., Nakajima T., Nakamura H., Arakawa T., Shiono H., Minato M. (2009). Acoustic emission measurement using a strain-insensitive fiber Bragg grating sensor under varying load conditions. Opt. Lett..

[8] Guo J., Yang C. (2015). Highly Stabilized Phase-Shifted Fiber Bragg Grating Sensing System for Ultrasonic Detection. IEEE Photonics Technol. Lett..

[9] Guan B.O., Tam H.Y., Lau S.T., Chan H.L.W. (2005). Ultrasonic hydrophone based on distributed Bragg reflector fiber laser. IEEE Photonics Technol. Lett..

[10] Liu T., Hu L., Han M. (2014). Adaptive ultrasonic sensor using a fiber ring laser with tandem fiber Bragg gratings. Opt. Lett..

[11] Wu Q., Okabe Y. (2012). Ultrasonic sensor employing two cascaded phase-shifted fiber Bragg gratings suitable for multiplexing. Opt. Lett..

[12] Minardo A., Cusano A., Bernini R., Zeni L., Giordano M. (2005). Response of fiber Bragg gratings to longitudinal ultrasonic waves. IEEE Trans. Ultrason. Ferroelectr. Freq. Control.

[13] Pierce S.G., Philp W.R., Gachagan A., McNab A., Hayward G., Culshaw B. (1996). Surface-bonded and embedded optical fibers as ultrasonic sensors. Appl. Opt..

[14] Wang D.H., Jia P.G., Wang S.J., Zhao C.L., Zeng D.P., Wang H., Li F.Q. (2013). Tip-sensitive all-silica fiber-optic Fabry–Perot ultrasonic hydrophone for charactering high intensity focused ultrasound fields. Appl. Phys. Lett..

[15] Zhang J.L., Sheng X.Z., Wu C.Q., Zhang L.J. Laser ultrasound detecting experiment with fiber Michelson interferometer. Proceedings of the Optical Fiber Communication and Optoelectronics Conference (IEEE).

[16] Jang T.S., Lee S.S., Kwon Il.B., Lee W.J., Lee J.J. (2002). Noncontact detection of ultrasonic waves using fiber optic Sagnac interferometer. IEEE Trans. Ultrason. Ferroelectr. Freq. Control.

[17] Beard P.C., Mills T.N. (1997). Miniature optical fibre ultrasonic hydrophone using a Fabry–Perot polymer film interferometer. Electron. Lett..

[18] Jiang J., Zhang T., Wang S., Liu K., Li C., Zhao Z., Liu T. (2017). Non-contact ultrasonic detection in low-pressure carbon dioxide medium using high sensitivity fiber-optic Fabry–Perot sensor system. J. Lightwave Technol..

[19] Morris P., Hurrell A., Shaw A., Zhang E., Beard P. (2009). A Fabry-Pérot fiber-optic ultrasonic hydrophone for the simultaneous measurement of temperature and acoustic pressure. J. Acoust. Soc. Am..

[20] Bae H., Yu M. (2012). Miniature Fabry–Perot pressure sensor created by using UV-molding process with an optical fiber based mold. Opt. Express.

[21] Liao C., Liu S., Liu S., Xu L., Wang C., Wang Y., Li Z., Wang Q., Wang D.N. (2014). Sub-micron silica diaphragm-based fiber-tip Fabry–Perot interferometer for pressure measurement. Opt. Lett..

[22] Wang Y., Wang D.N., Wang C., Hu T. (2013). Compressible fiber optic micro-Fabry-Pérot cavity with ultra-high pressure sensitivity. Opt. Express.

[23] Sun B., Wang Y., Qu J., Liao C., Yin G., He J., Zhou J., Tang J., Liu S., Li Z. (2015). Simultaneous measurement of pressure and temperature by employing Fabry–Perot interferometer based on pendant polymer droplet. Opt. Express.

[24] Guo F., Fink T., Han M., Koester L., Turner J., Huang J. (2012). High-sensitivity, high-frequency extrinsic Fabry–Perot interferometric fiber-tip sensor based on a thin silver diaphragm. Opt. Lett..

[25] Xu F., Ren D., Shi X., Li C., Lu W., Lu L., Lu L., Yu B. (2012). High-sensitivity Fabry–Perot interferometric pressure sensor based on a nano-thick silver diaphragm. Opt. Lett..

[26] Gang T., Hu M., Rong Q., Qiao X., Liang L., Liu N., Tong R., Liu X., Bian C. (2016). High-Frequency Fiber-Optic Ultrasonic Sensor Using Air Micro-Bubble for Imaging of Seismic Physical Models. Sensors.

[27] Ma J., Ju J., Jin L., Jin W.W. (2011). A compact Fiber-Tip micro-cavity sensor for high-pressure measurement. IEEE Photonics Technol. Lett..

[28] Dakin J.P., Ecke W., Schroeder K., Reuter M. (2009). Optical fiber sensors using hollow glass spheres and CCD spectrometer interrogator. Opt. Lasers Eng..

[29] Bar-Zion A., Tremblay-Darveau C., Solomon O., Dan A., Eldar Y.C. (2016). Fast vascular ultrasound imaging with enhanced spatial resolution and background rejection. IEEE Trans. Med. Imaging.

